# Tubo-Ovarian Abscess (with/without Pseudotumor Area) Mimicking Ovarian Malignancy: Role of Diffusion-Weighted MR Imaging with Apparent Diffusion Coefficient Values

**DOI:** 10.1371/journal.pone.0149318

**Published:** 2016-02-19

**Authors:** Tingting Wang, Wenhua Li, Xiangru Wu, Bing Yin, Caiting Chu, Ming Ding, Yanfen Cui

**Affiliations:** 1 Department of Radiology, Xinhua Hospital affiliated to Shanghai Jiao Tong University School of Medicine, 1665 Kong Jiang Road, Shanghai 200092, China; 2 Department of Pathology, Xinhua Hospital affiliated to Shanghai Jiao Tong University School of Medicine, 1665 Kong Jiang Road, Shanghai 200092, China; University of Chicago, UNITED STATES

## Abstract

**Objective:**

To assess the added value of diffusion-weighted magnetic resonance imaging (DWI) with apparent diffusion coefficient (ADC) values compared to MRI, for characterizing the tubo-ovarian abscesses (TOA) mimicking ovarian malignancy.

**Materials and Methods:**

Patients with TOA (or ovarian abscess alone; n = 34) or ovarian malignancy (n = 35) who underwent DWI and MRI were retrospectively reviewed. The signal intensity of cystic and solid component of TOAs and ovarian malignant tumors on DWI and the corresponding ADC values were evaluated, as well as clinical characteristics, morphological features, MRI findings were comparatively analyzed. Receiver operating characteristic (ROC) curve analysis based on logistic regression was applied to identify different imaging characteristics between the two patient groups and assess the predictive value of combination diagnosis with area under the curve (AUC) analysis.

**Results:**

The mean ADC value of the cystic component in TOA was significantly lower than in malignant tumors (1.04 ± 0 .41 × 10^−3^ mm^2^/s vs. 2.42 ± 0.38 × 10^−3^ mm^2^/s; p < 0.001). The mean ADC value of the enhanced solid component in 26 TOAs was 1.43 ± 0.16×10^−3^mm^2^/s, and 46.2% (12 TOAs; pseudotumor areas) showed significantly higher signal intensity on DW-MRI than in ovarian malignancy (mean ADC value 1.44 ± 0.20×10^−3^ mm^2^/s vs.1.18 ± 0.36 × 10^−3^ mm^2^/s; p = 0.043). The combination diagnosis of ADC value and dilated tubal structure achieved the best AUC of 0.996. Sensitivity, specificity, positive predictive value (PPV), negative predictive value (NPV), and accuracy of MRI vs. DWI with ADC values for predicting TOA were 47.1%, 91.4%, 84.2%, 64%, and 69.6% vs. 100%, 97.1%, 97.1%, 100%, and 98.6%, respectively.

**Conclusions:**

DW-MRI is superior to MRI in the assessment of TOA mimicking ovarian malignancy, and the ADC values aid in discriminating the pseudotumor area of TOA from the solid portion of ovarian malignancy.

## Introduction

Clinically, a patient with acute pelvic infection typically presents with fever, chills, leukocytosis and/or increased blood C-reactive protein (CRP), which is distinct from adnexal malignant tumor. However, previous reports suggest that approximately 50% of women with chronic infections such as TOA, can have normal body temperature and laboratory examination results [1]. Other symptoms and signs including abdominal pain, vaginal discharge and abnormal vaginal bleeding are nonspecific for TOA and malignancy [1–2].

In cases of complicated TOAs, purulent cavities, thickened walls or septa, and adjoining dilated fallopian tube (salpingitis or pyosalpinx), are usually demonstrative of large multilocular cystic lesions or mixed cystic and solid masses on magnetic resonance imaging (MRI) [1,3]. However, MRI findings of thickened wall or septa (> 3 mm) or various degrees of solid portions are strongly suggestive of ovarian malignancy [4–6]. The pus can also present pseudosolid-like or mucin-like signal intensity on T1- and T2-weighted images, which can mimic soft-tissue or the mucinous components of epithelial ovarian tumors [7]. Additionally, inflammation can often cause damage to the rectosigmoid colon and ureter, leading to reactive lymph node swelling and the production of moderate to considerable free fluid in the pelvis. These MRI findings associated with vague clinical symptoms of infection can further contribute to errors in diagnosis, and suspicion of ovarian malignancy [1].

TOA is a serious acute complication of pelvic inflammatory disease (PID). Approximately 30% of patients with PID develop TOA, which can begin insidiously without fever or acute abdominal pain. Timely administration of antibiotics or interventional drainage are crucial for a patient’s reproductive function. It is therefore imperative for clinicians to identify patients with TOA and differentiate it from ovarian malignancy, in order to obviate unnecessary resection or chemotherapy [8–9].

Diffusion-weighted magnetic resonance imaging (DWI) and the calculated apparent diffusion coefficient (ADC) are widely used to distinguish necrotic or cystic brain tumors from abscesses [10–11]. Recent studies have demonstrated that DWI, with measurement of ADC values, can be a useful tool in the diagnosis of abdominal, musculoskeletal and soft-tissue abscesses, and effective in characterizing pus, serous fluid, mucinous fluid and blood components in pelvic inflammatory disease (PID) and non-PID [8,12–15]. While some reports have described DWI and ADC values for differentiating pelvic abscesses from pelvic cystic tumors [16–17], there is very little data available on the assessment of complicated TOAs mimicking ovarian malignancy, and discriminating the enhanced solid area of TOA with high signal intensity on DWI (pseudotumor area) from the solid portion of ovarian malignancy.

In the present study, we evaluated both the cystic and solid component of TOAs and ovarian malignant tumors using DWI and the ADC values. The purpose of this study was to determine whether DWI and ADC values are able to characterize TOAs (with/without pseudotumor area) mimicking ovarian malignancy.

## Materials and Methods

### Patient selection

The study was approved by Ethics Committee of Xinhua Hospital Affiliated to Shanghai Jiaotong University School of Medicine, Shanghai, China. Patient information was anonymized prior to analysis, and no specimens or tissue samples were collected from patients, and hence the requirement for informed consent was waived. The study evaluated all the patients admitted to our hospital between January 2010 and February 2015 with suspected TOA or ovarian malignant tumors on the basis of clinical symptoms or signs, laboratory examination results, ultrasound or computer tomography (CT) analysis. All subjects received both MRI and DWI within the institute, and the final diagnoses were confirmed by pathological examination or culture of aspiration material. The subjects’ data were collected between September 2014 and June 2015, and authors had access to identifying information during and after data collection.

Patients with endometrioma (n = 17), mature teratoma (n = 19), cystadenofibroma (n = 10), fibrothecoma and fibroma (n = 32), subserosal leiomyoma (n = 9), pyosalpinx and salpingitis (n = 12), secondary adnexal tumor (n = 17) and DWI artifacts (n = 3) were excluded to limit selection bias. The cases of complete solid mass with no cystic components (n = 3) including ovarian malignant mixed Mullerian tumor, ovarian endometrial adenocarcinoma, ovarian clear cell carcinoma, were also excluded because they were distinct findings.

The final cohort included 69 patients (premenopausal 26 and postmenopausal 43; mean age 47.4 years; age range 21–85 years). This included 45 TOAs (or ovarian abscesses) in 34 patients, and 37 ovarian malignant tumors in 35 patients. The 35 cases of ovarian malignancy included borderline cystoadenoma (n = 8), cystoadenocarcinoma (n = 12), endometrial adenocarcinoma (n = 5), clear cell carcinoma(n = 7), granulosa cell tumor (n = 3; Table 1).

**Table 1:** Pathology correlated to gross MRI morphology.

Disease	Patient No. ^a^	Lesions	Group A^b^	Group B^b^
**TOA (or ovarian abscess)**	**34**	Unilateral 23; bilateral 11	**8**	**26**
**Malignancy**	**35**	Unilateral 33; bilateral 2	**9**	**26**
Borderline cystoadenomas	8	Unilateral 8	6	2
Cystoadenocarcinomas	12	Unilateral 12	3	9
Clear cell carcinoma	7	Unilateral 5; bilateral 2	0	7
Endometrial adenocarcinoma	5	Unilateral 5	0	5
Granulosa cell tumor	3	Unilateral 3	0	3

^a^No. = number.

^b^Group A = predominantly cystic mass with thicken wall or internal septation; Group B = mixed cystic and solid mass.

All patients complained of lower abdominal pain or mass. Inflammatory symptoms and laboratory examination results (fever, elevated CRP, elevated leukocyte count), and other signs and symptoms including abdominal pain, vaginal discharge, abnormal vaginal bleeding and tumor marker (CA125), were recorded.

### Imaging protocols

MRI was performed on 3.0-T MR scanners (Signa, GE Medical Systems, Milwaukee, WI,USA) using a torso phase array coil. The imaging protocol was comprised of axial noncontrast T1-weighted and axial T2-weighted imaging(TR/TE range 400–600/10–14 ms and 4000–6000/100–120 ms, respectively). Imaging was performed with a chemical shift-selective fat saturation pulse using the following parameters: slice thickness of 6 mm; gap of 1 mm; field of view (FOV) 32–42 cm; matrix 256×256; and excitation at 2. Sagittal T1-weighted and T2-weighted fast spin-echo imaging (TR/TE range 3000–6000/100–110 ms) without chemical shift-selective fat saturation pulse was also performed, as well as post-contrast enhanced axial and sagittal T1-weighted imaging, using the same parameters described above.

DWI was acquired in the axial plane prior to administration of contrast medium, by using a single shot echo-planar imaging sequence (TR/TE effective range 8000–10000/70–100; slice thickness of 6 mm; gap of 1 mm; FOV 32–42 cm; matrix 128×128; excitation at 2). B values of 0 and 1000 s/mm^2^ were also applied in three orthogonal (Z, Y, and X) directions.

### Image analysis

MRI data were analyzed by two radiologists (who had 11 and 12 years of experience in pelvic MRI, respectively). The reviewers were blinded to the clinical presentation and laparoscopic and pathological diagnosis of the lesion and assessed all images together, in two steps. Firstly, the radiologists reviewed the MRI images. In the second step, they reviewed the MRI and DWI images. For both steps, the observers characterized lesions as TOA (or ovarian abscess) or malignancy. Discrepancies in interpretation were resolved by consensus, and a final diagnosis was made by two radiologists. On MRI, signal intensity characteristics and morphological appearance of lesions were evaluated, including lesion size, shape, character (cystic and solid component), signal intensity of cystic and solid components on T1-weighted, T2-weighted and DW images. The normal outer myometrium was used as a signal intensity reference on T1-weighted, T2-weighted and DW images (grade 1: lower than myometrium; grade 2: parallel to myometrium; grade 3: higher than myometrium).

A tubo-ovarian or ovarian abscess was defined as an ill-defined adnexal mass with thick regular or irregular enhanced walls containing fluid. The signal intensity was variable from low signal intensity on T1-weighted images to intermediate or high signal intensity on T2-weighted images. The solid component according to a previously established classification by Timmerman et al. included thickened septa or wall, vegetation (papillary projection), and various degrees of solid portions, which showed enhancement after injection. The cystic component was defined as tissue that had homogeneous long T1 and T2 characteristics or different signal intensities on T1-or T2-weighted MR images and showed no enhancement after injection.

All ADC measurements were made by one radiologist, using an Advantage Windows workstation 4.2 (GE Healthcare, Milwaukee, WI, USA). On the ADC maps, more than three circular regions of interest (ROIs) were placed at targeted areas in the cystic and/or solid components of the lesions, by referring to MRI images. To eliminate the potential influence of multiple comparisons in the same subject who had bilateral lesions (n = 13), only the results for the largest and most complex lesion were considered. The cystic lesions with apparent hemorrhage on T1-weighted images were excluded from the ROI measurement. To minimize variability, the final ADC values of each patient were determined by the average results of all measured ROIs.

### Statistical analysis

Laparoscopic and pathological findings or culture of aspiration material were used as the reference standard. Quadratic K coefficients were calculated to assess the interobserver agreement between MRI and combined MRI and DWI readers, with regard to lesion characterization. A k-value of 0.61–0.80 was indicative of substantial agreement, and a value of 0.80 reflected almost perfect agreement. For comparisons of the categorical variables between the two groups, the Chi-squared and Fisher Exact tests were employed. The Student t-test was used to analyze the differences in age and ADC values between the two groups. The Mann-Whitney U test was used to compare the tumor size, and the thickness of the wall in the two groups. A binary, multivariate logistic regression analysis was performed with selected variables to identify imaging characteristics that differed between the two groups.The area under ROC curve (AUC) was applied to assessed the diagnostic efficacy. Statistical calculations were performed using SPSS for Windows version 19.0 (SPSS, Chicago, IL, USA). Findings with a p-value < 0.05 were considered statistically significant.

## Results

### Pathological and morphological findings

According to the gross morphology and internal structure, all cases were divided into two groups: Group A, which is predominantly cystic mass with thickened wall or internal septation, and Group B, which is mixed cystic and solid mass (Table 1).

In the TOA (or ovarian abscess) category (n = 34), 8 cases were cystic masses with thickened walls or internal septation (Group A), and 26 cases had mixed cystic and solid masses (Group B). In the malignant category (n = 35), 6 borderline cystoadenomas and 3 cystoadenocarcinomas were characterized as Group A, and the remaining were assigned into Group B (which included all ovarian clear cell carcinomas, endometrial adenocarcinomas and granulosa cell tumors).

### Baseline characteristics

The clinical characteristics for patients with TOA(or ovarian abscess) or ovarian malignancy are summarized in Table 2.

**Table 2:** Clincal characteristics of TOA (or ovarian abscess) and ovarian malignancy.

Variables	TOA(or ovarian abscess)	Malignancy	p-value
Age (years)	40.9 ± 9.9	53.8 ± 13.2	0.452
**Inflammatory symptoms**			
Fever	20/34 (61.8%)	2/35 (5.7%)	<0.001
Abdominal pain	29/34 (85.3%)	14/35 (40%)	<0.001
Vaginal discharge	11/34 (32.4%)	5/35 (14.3%)	0.075
**Laboratory examination**			
Elevated leukocyte count	12/34 (35.3%)	4/35 (11.4%)	0.039
Elevated blood C-reactive protein (>10 mg/L)	18/34 (52.9%)	8/35 (22.9%)	0.010
**Tumor marker**			
CA125 (>35 UI/L)	21/34 (61.8%)	19/35 (54.3%)	0.529

The mean age of patients in the TOA(or ovarian abscess) category (40.9 ± 9.9 years) was less than that of the malignant category (53.8 ± 13.2 years), though there was no significant difference. Compared to the malignancy group, the presentation of fever and abdominal pain were significantly more common in the TOA group (p < 0.001), except for the symptom of vaginal discharge. There was significant difference between the two groups for the elevated leukocyte count (p = 0.039) and blood CRP (>10 mg/L; p = 0.010). No significant differences were observed in the elevation of tumor marker, CA125 (>35 U/mL) between the two groups.

### MRI features

MRI findings for patients with TOA and ovarian malignancy are summarized in Table 3.

**Table 3:** MR imaging findings of TOA and ovarian malignancy.

The features of MR imaging findings	TOA	Malignancy	p-value
**Characteristic**			
Tumor size(cm)	6.8 ± 2.5	10.7 ± 6.3	0.001
Indistinct margin	33/34 (97.1%)	16/35 (45.7%)	<0.001
Involvement	33/34 (97.1%)	15/35 (42.9%)	<0.001
Tubal structures	23/34 (67.6%)	5/35 (14.3%)	<0.001
Fluid-fluid level	6/34 (17.6%)	4/35 (11.4%)	0.695
Moderate or massive ascites	3/34 (8.8%)	6/35 (17.1%)	0.305
Soft tissue or pseudosolid signal intensity on T1/T2WI^a^	28/34 (82.4%)	31/35 (88.6%)	0.695
**Group A**	**TOA**	**Malignancy**	**p-value**
Thickened wall and/or septa(≥3mm)	6.4 ± 3.6	6.7 ± 4.5	0.670
Number of cysts			0.153
Unilocular	1 (12.5%)	1 (11.1%)	
Multilocularity (≤10)	7 (87.5%)	3 (33.3%)	
Multilocularity (≥10)	0 (0%)	5 (55.6%)	
**Group B**	**TOA**	**Malignancy**	**p-value**
Morphology of solid portion			<0.001
Projections or nodules	2 (7.7%)	14 (53.8)	
Solid and cystic with multilocularity	3 (11.5%)	6 (23.1%)	
Mass with central cystic changes	21 (80.8%)	6 (23.1%)	

^a^T1/T2WI = T1-weighted image and (or) T2-weighted image.

The size of the tumors in the ovarian malignancy group were significantly larger than the abscesses in the TOA group (10.7 ± 6.3cm vs. 6.8 ± 2.5cm, respectively; p = 0.001). Compared to the ovarian malignant tumors, TOAs more frequently showed indistinct margins and invasion into adjacent organs (p <0.001). Association with tubal dilatation was more common in TOA than in ovarian malignancy (67.6% vs. 14.3%, respectively; p <0.001). However, there were no significant differences between the two groups for fluid-fluid level and ascites. Both TOA and ovarian malignancy can demonstrate soft-tissue or pseudosolid signal intensity on T1/T2WI MRI, and no significant differences were observed between them.

In group A, there was no statistically significant difference in the thickness of enhancing wall and/or septa between the TOA and ovarian malignancy. Both TOA and malignancy in group A showed unilocularity (12.5% vs. 11.1%, respectively), and none of the TOAs showed multilocularity with >10 cavities. TOAs more frequently showed multilocular cystic lesion with <10 cavities than ovarian malignancies, which typically presented honeycomb-like lesions consisting of >10 cavities. However, these differences were not statistically significant.

Considering the morphology of solid portion in groupB, statistically significant differences between TOA and malignancy (p < 0.001). Projections or nodules (53.8%), as well as solid and cystic masses with multilocularity (23.1%) were observed more often in ovarian malignancy, while masses with central cystic changes were more common in TOAs (80.8%).

### DWI findings and the ADC values

Table 4 shows DWI features and ADC values in TOAs and ovarian malignancy.

**Table 4:** DWI features and ADC values between TOA and ovarian malignancy.

	TOA	Malignancy	p-value
**DWI within cystic component**			<0.001
Low or intermediate signal intensity	0 (0%)	31 (88.6%)	
High signal intensity	34 (100%)	4 (11.4%)	
**DWI within solid component in group B**			<0.001
Low or intermediate signal intensity	14 (53.8%)	0 (0%)	
High signal intensity	12 (46.2%)	26 (100%)	
**Cystic ADC**	1.04 ± 0.41	2.42 ± 0.38	<0.001
**Solid ADC in group B**	1.43 ± 0.16	1.18 ± 0.36	0.002

**Fig 1:**
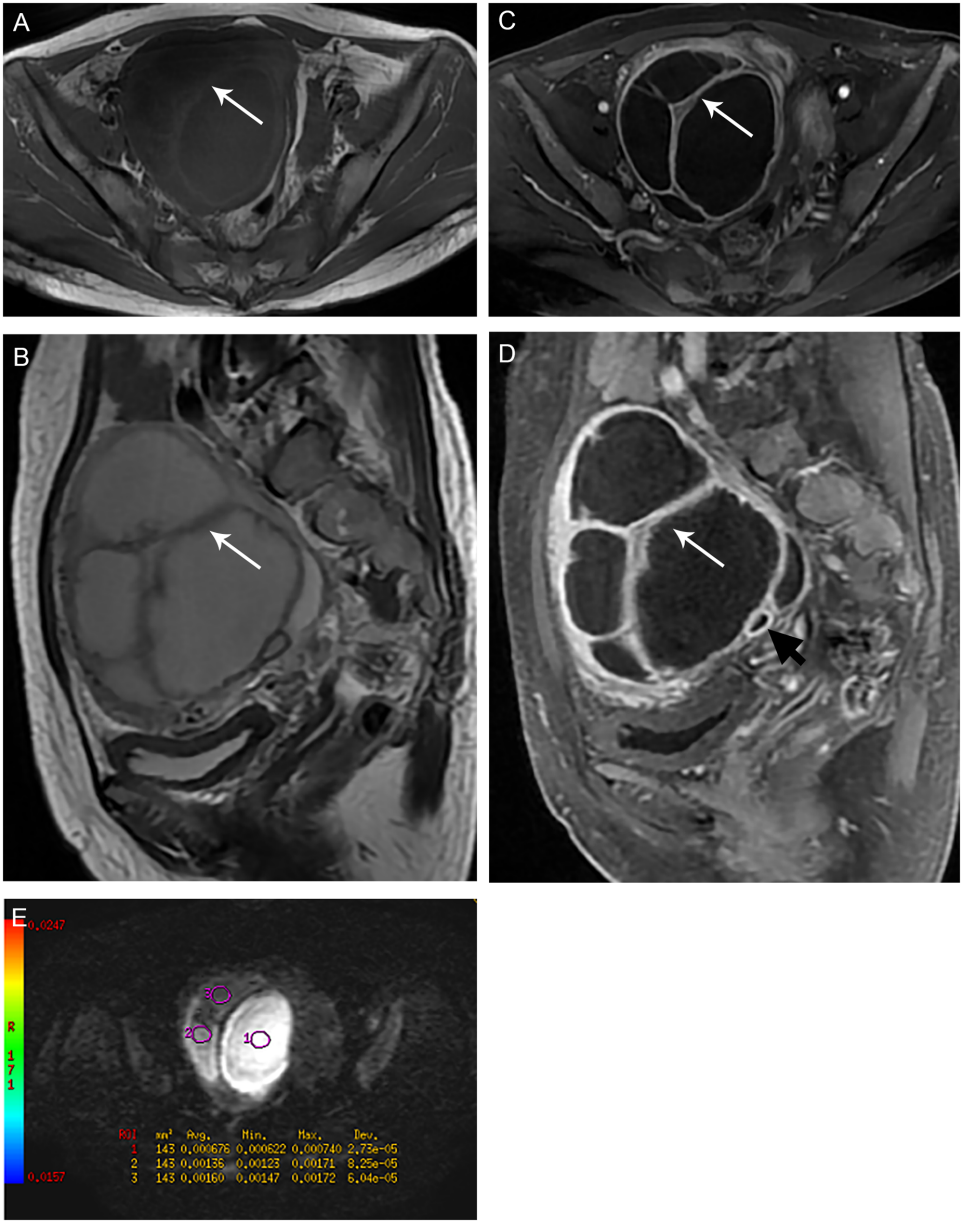
Group A TOA: 48-year-old woman with right ovarian abscess, showing a predominantly cystic appearance. (A) Axial T1-weighted image shows a large multilocular cystic mass with high signal intensity of the inner walls (white arrow). (B) Sagittal T2-weighted image shows a mainly cystic mass with thick, irregular septa and wall. The contents of cavities have high signal intensity, which is lower than that of a pure cyst. (C-D) Axial and sagittal contrast-enhanced MR images show a multilocular cystic mass with enhancing thick walls and septa, along with a few adjacent tubal structures(hydrosalpinx, black arrow). (E) Axial DW image shows that the fluid collections have heterogeneous high and intermediate signal intensity with different ADC values, corresponding to different states of pus with various degree of water diffusion restriction.(circle 1 ADC = 0.676 × 10^−3^ mm^2^/s, circle 2 ADC = 1.360 × 10^−3^ mm^2^/s, circle 3 ADC = 1.60 × 10^−3^ mm^2^/s).

All 34(100%) cases of TOA showed markedly homogeneous or heterogeneous hyperintensity in the cystic componant on DW images. Corresponding ADC maps of the cystic portion showed hypointensity, indicating restricted water diffusion (Fig 1). The cystic component of all malignant tumors exhibited hypointensity on DWI. The mean ADC value of the cystic component in the TOAs and malignant tumors were 1.04 ± 0.41 × 10^−3^ mm^2^/s and 2.42 ± 0.38 × 10^−3^ mm^2^/s, respectively (p < 0.001). Four (11.4%) women with ovarian malignancy showed fluid-fluid level with hyperintensity of the debris and hypointensity of the fluid on DWI. ADC maps also showed hypointensity of the debris, which may be characterized as hemorrhage by MRI.

**Fig 2:**
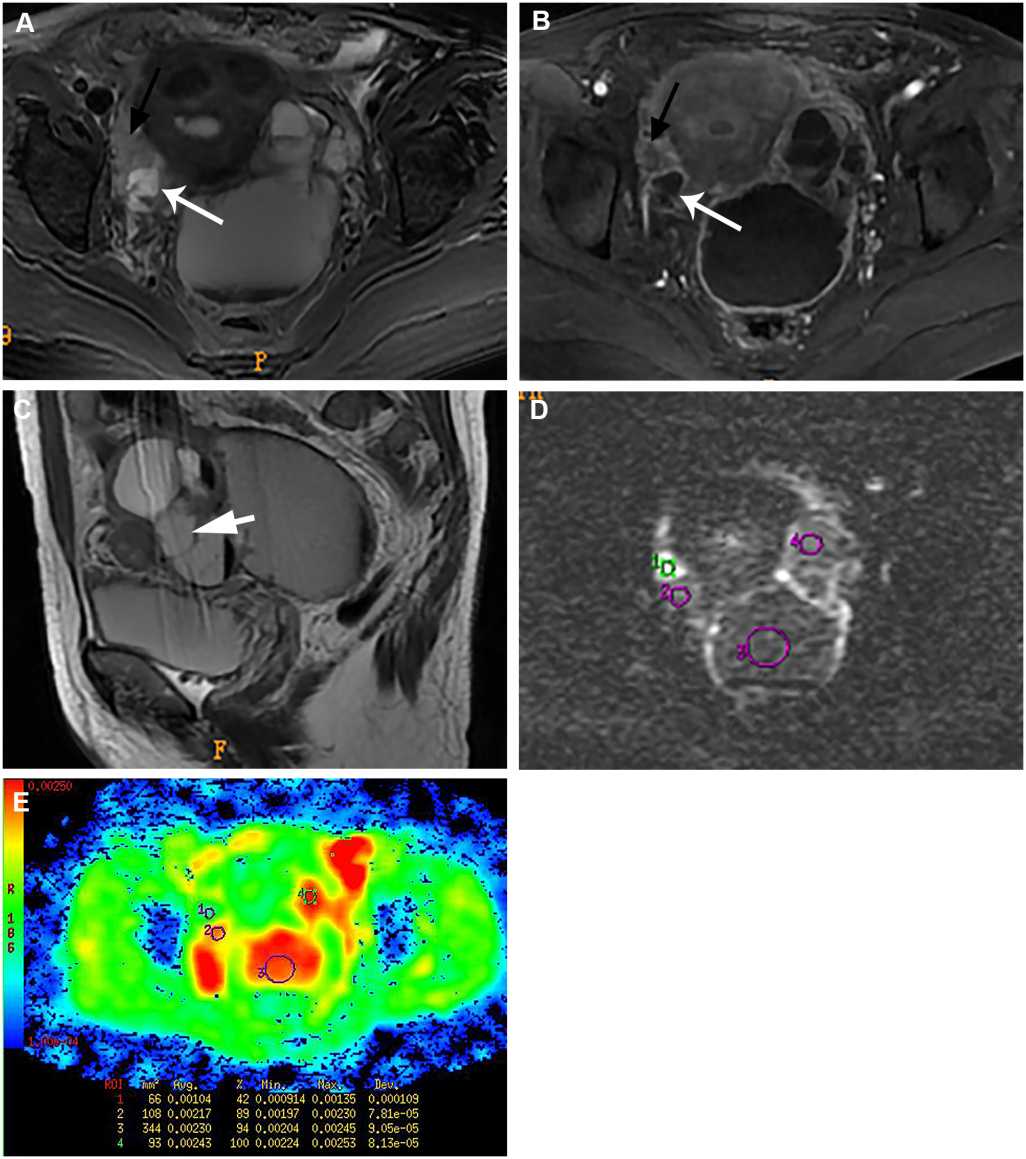
Groups A and B, ovarian malignancy: A 47-year-old woman with bilateral ovarian serous cystadenocarcinoma. The left adnexal mass shows a predominantly cystic appearance (Group A), while the right mass has a mixed cystic and solid appearance (Group B). (A) Axial T2-weighted image shows heterogeneous signal intensity within the bilateral adnexal masses with indistinct margin. (B) Axial contrast-enhanced MR image shows a mixed cystic and solid mass in right pelvic cavity, with the solid portion strikingly enhanced (long black arrow). The left adnexal mass shows a predominantly cystic lesion with thick, irregular enhencing wall and the adjacent dilated tube. (C) The adjacent dilated fallopian tube(short white arrow) and fluid-fluid level are visible within the left pelvic mass. (D-E) Axial DW images and corresponding ADC map show that the solid portion is hyperintensity with a low ADC value (circle 1 ADC = 1.04 × 10^−3^ mm^2^/s). All the cystic components show high signal intensity on DW images with high ADC values indicating no water diffusion restriction (circles 1–3: ADC = 2.170 × 10^−3^ mm^2^/s, 2.300 × 10^−3^ mm^2^/s, 2.430 × 10^−3^ mm^2^/s, respectively).

**Fig 3:**
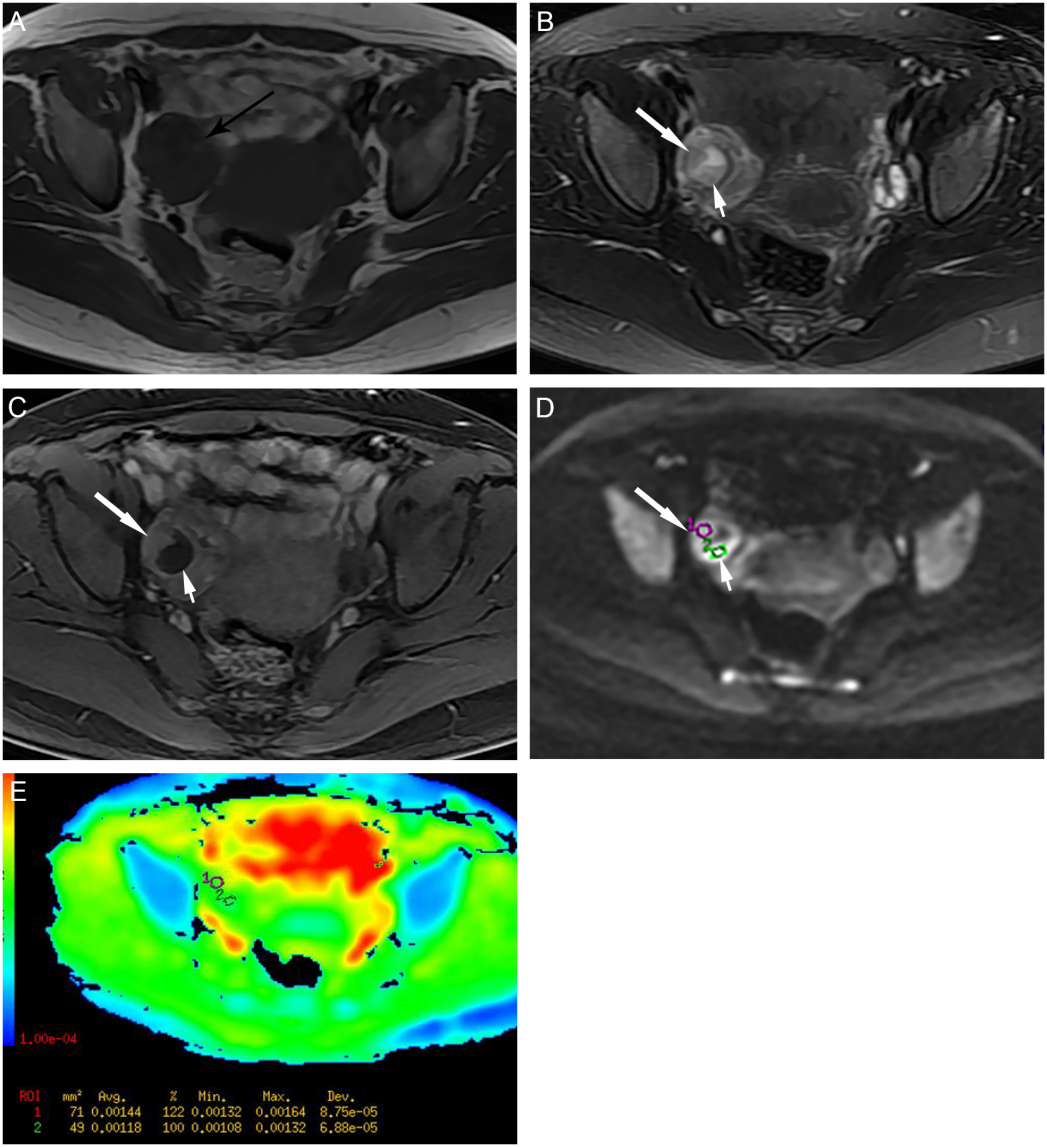
A 37-year-old woman with right ovarian abscess, showing a complex mixed cystic and solid appearance. (A) Axial T1-weighted image shows a intermediate signal intensity mass in the right side of the pelvic cavity. (B) Axial T2-weighted image indicates that the mass has intermediate signal intensity and a central cystic component (high signal intensity area). (C) Axial contrast-enhanced MR image shows a mixed cystic and solid mass with thick wall, presenting marked rim-enhancement. The soft-tissue signal intensity area (pseudosolid) on the T2-weighted image shows no enhancement (short white arrow). (D-E) Axial DW image shows the pseudosolid area is high signal intensity, compatible with purulent cavities with low ADC value. The enhancing solid component (long white arrow) also shows high signal intensity on DW image, mimicking an adnexal maligancy (pseudotumor area), with a relatively high ADC value (circle 1 ADC = 1.440 × 10^−3^ mm^2^/s; circle 2 ADC = 1.180 × 10^−3^ mm^2^/s).

**Fig 4:**
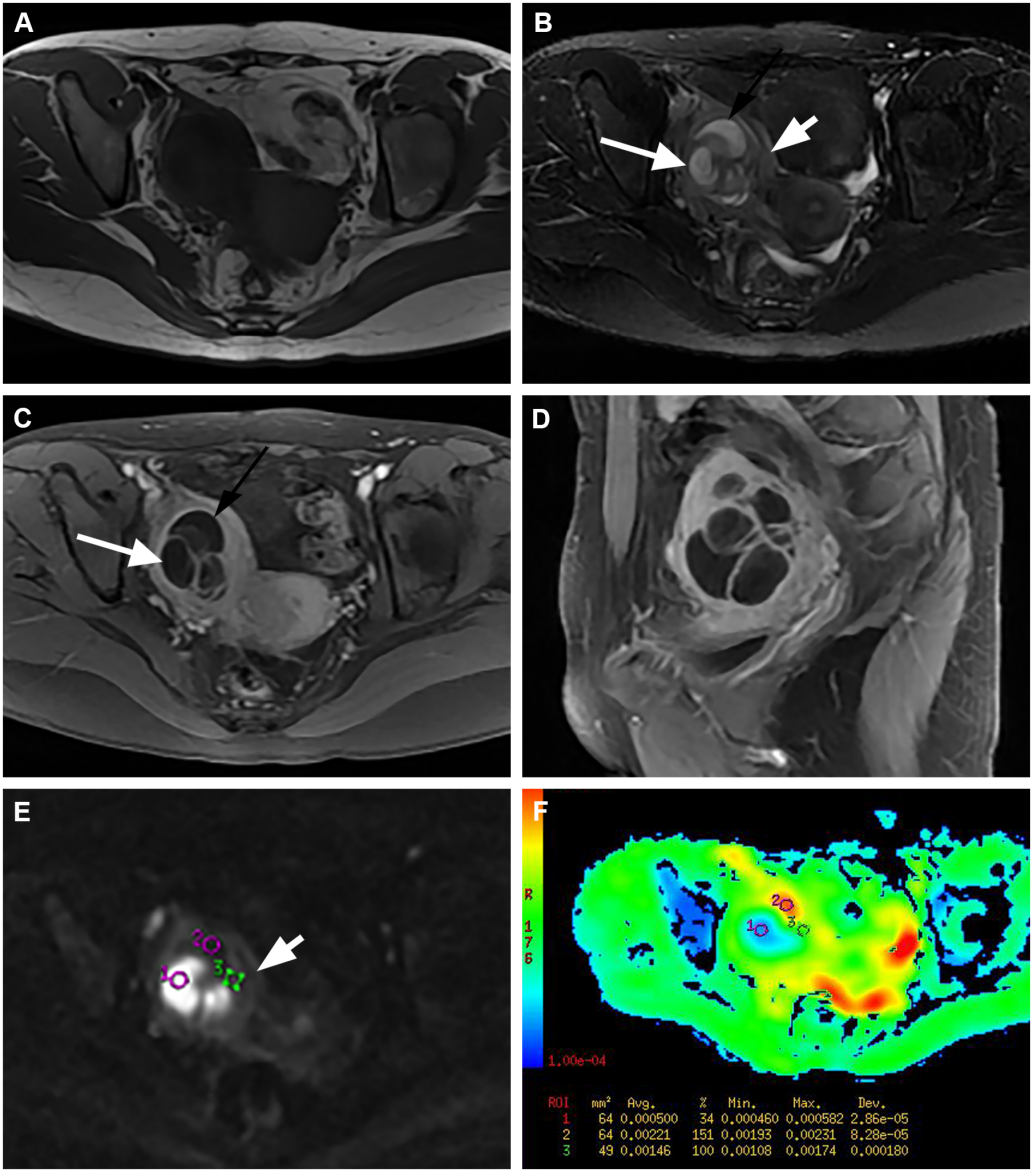
Group B TOA: A 24-year-old woman with right ovarian abscess, showing a mixed cystic and solid appearance suspicious for ovarian malignancy. (A) Axial T1-weighted image shows a low heterogeneous signal intensity mass in the right adnexal region. (B) Axial T2-weighted image shows a heterogeneous signal intensity mass, with some regions of high signal intensity (black and white arrows). (C-D) Axial and sagittal contrast-enhanced MR images show a complex cystic and solid mass with marked enhancement of the solid component, and thick, irregular walls and septa. The ill-defined mass involves the myometrium and potentially rectosigmoid bowel wall. (E-F) Axial DW images and the corresponding ADC map shows that the solid portion is hypointensity with a high ADC value (short white arrow; circle 3 ADC = 1.460 × 10^−3^ mm^2^/s); The cystic portions which show hyperintensity on the T2-weighted image, show different signal intensities on DW images: high signal intensity compatible with pus with a low ADC value (white arrow) and low signal intensity indicating the fluid with no restricted water diffusion (black arrow; circle 1 ADC = 0.500 × 10^−3^ mm^2^/s; circle 2 ADC = 2.210 × 10^−3^ mm^2^/s).

In contrast, the enhanced solid components of all 26 malignant tumors showed high signal intensity on DWI, with the mean ADC value of 1.18±0.36 × 10^−3^ mm^2^/s on the corresponding ADC maps (Fig 2). Twelve (46.2%) of the 26 TOAs in group B showed high signal intensity in the solid component on DWI (pseudotumor area; Fig 3), while the remaining 53.8% showed low or intermediate signal intensity (Fig 4). The mean ADC value of the solid component in TOA was 1.43±0.16×10^−3^mm^2^/s, which was significantly higher than that in ovarian malignancy (p = 0.002).

There are many overlaps between the ADC values of cystic and solid components of TOA and malignancy, especially for those with high signal intensity on DWI (Table 5). The mean ADC values did not differ significantly between the cystic portions of TOA and solid portions of ovarian malignancy. However, solid portions with high DWI in TOA were significantly higher than mean ADC values of the cystic portions of TOA (p = 0.043) and the solid portions of ovarian malignancy (p = 0.002). Table 6 shows the diagnostic predictive values for assessment of TOA and ovarian malignancy for each technique and reviewer.

**Table 5:** ADC values of high signal intensity areas on DWI.

High signal intensity area on DWI	Number	Mean ADC	p-value
Cystic portion of TOA	34	1.04 ± 0.41	
Solid portion of malignancy	26	1.18 ± 0.36	
Solid portion with high DWI of TOA	12	1.44 ± 0.20	
Cystic portion of TOA vs solid portion of malignancy			0.152
Solid portion with high DWI of TOA vs solid portion of malignancy			0.043
Solid portion with high DWI of TOA vs cystic portion of TOA			0.002

**Table 6:** Differential performance between TOA and ovarian malignancy.

Parameter	MRI^b^	DMRI^b^
No.of TP^a^ findings	16	34
No.of TN^a^ findings	32	34
No.of FP^a^ findings	3	1
No.of FN^a^ findings	18	0
**Sensitivity (%)**	47.1	100
**Specificity (%)**	91.4	97.1
**PPV**^a^ **(%)**	84.2	97.1
**NPV**^a^ **(%)**	64	100
**Accuracy (%)**	69.6	98.6

^a^TP = true positive, TN = true negative, FP = false positive, FN = true negative, PPV = positive predictive value, NPV = negative predictive value.

^b^MRI = MR imaging, DMRI = MR imaging with DWI.

### Combined test assessed with ROC curve based on Logistic regression

**Fig 5:**
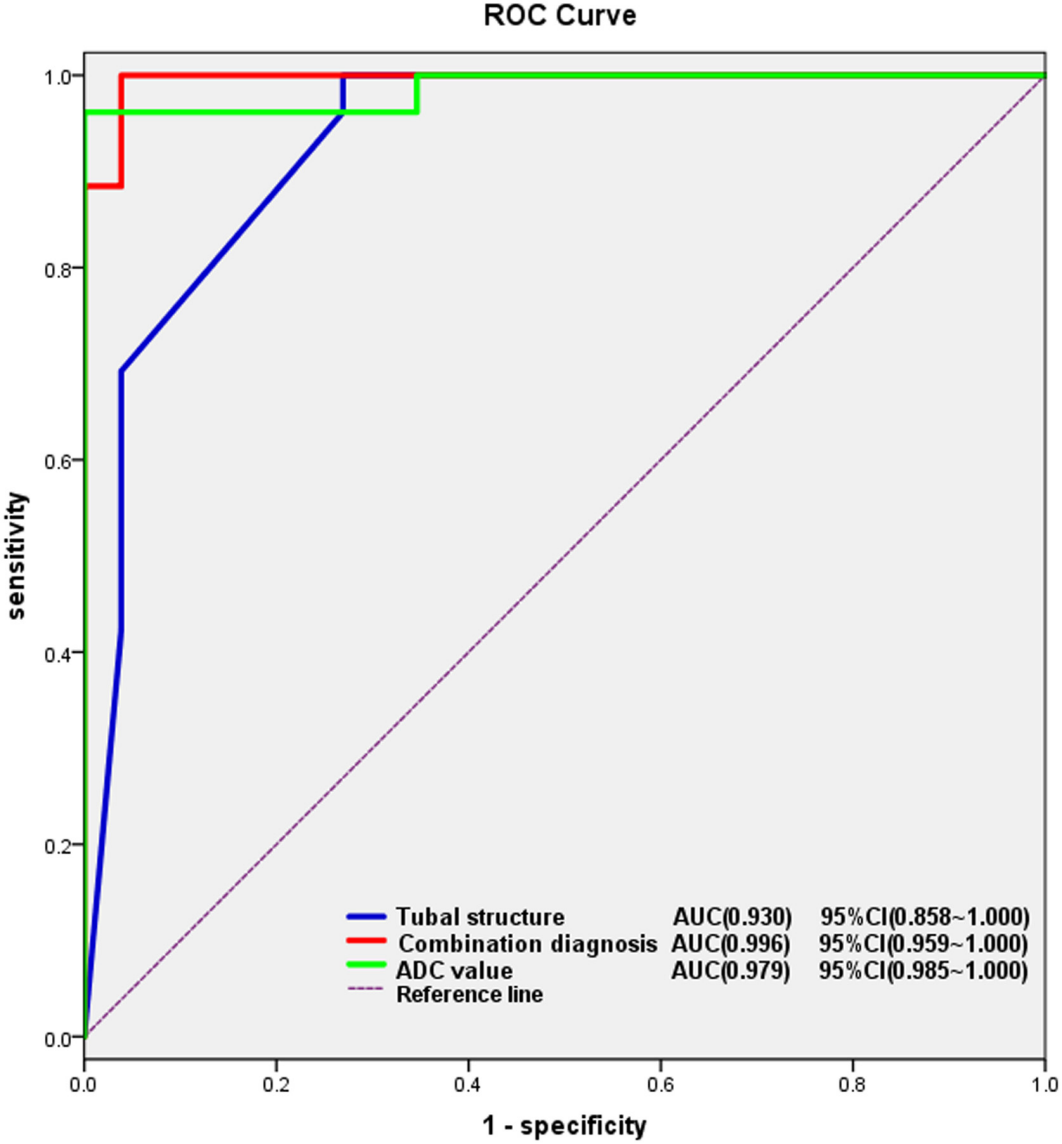
A ROC curve analysis of tubal structure, ADC value and a combination diagnosis. The ROC curve analysis shows combination diagnosis of ADC value and dilated tubal structure achieved the best AUC. The area under ROC curve (AUC) of the three parameters: AUC of combination diagnosis = 0.996 (95% CI: 0.959–1.000), AUC of tubal structure = 0.930 (95% CI: 0.858–1.000), AUC of ADC value = 0.979 (95% CI: 0.985–1.000).

In a multivariate logistic regression model, we screened multiple diagnostic parameters for TOA. We examined the following imaging features that differed between the two groups: size, indistinct margin, involvement, tubal structure, moderate or massive ascites, ADC value and morphology of solid portion. This analysis demonstrated that tubal structure and ADC value were statistically significant variables (p = 0.049, p = 0.002). Then, using the predicted probability of the combined model as the analyzed variable, we calculated the area under ROC curve (AUC) with the ROC curve to compare the diagnostic efficacy of combined determinations with other parameter alone. The ROC curve analysis shows the AUC of combination diagnosis (ADC value combined with tubal structure) was greater than that of tubal structure or ADC value alone (Fig 5).

### Comparison of MRI with combined MRI and DWI

MRI analyses were consistent with TOA diagnosis in 47.1% (16/34) and ovarian malignancy diagnosis in 91.4% (32/35) of the 69 patients. The sensitivity, specificity, positive predictive value (PPV), negative predictive value (NPV), and accuracy of MRI findings for predicting TOA were 47.1%, 91.4%, 84.2%, 64%, and 69.6%, respectively. When DWI was added to MR protocols, the sensitivity, specificity, PPV, NPV, and accuracy of MR imaging findings for predicting TOA were 100%, 97.1%, 97.1%, 100%, and 98.6%, respectively. Thus, the combined DWI imaging strategy may greatly increase accuracy, sensitivity, PPV and NPV of MRI for distinguishing TOA from ovarian malignancy. Moreover, there was comparatively good agreement between the radiologists in lesion characterization between MRI imaging and the combination of MRI with DWI (k = 0.624).

## Discussion

TOA is a severe complication of pelvic inflammatory disease (PID). If acute pelvic inflammation does not have timely diagnosis and adequate treatment, it may gradually progress into a TOA or a chronic inflammatory mass [3].

TOA are most prevalent in women aged 30–50 years, but rarely occur in childhood, adolescence or in the postmenopausal years. In the current study, patients diagnosed with TOA were at least a decade younger than those diagnosed with ovarian malignancy. Clinical imformation and laboratory examinations play a key role in diagnosis of TOA. Many common gynecologic complaints are nonspecific for TOA and other adnexal tumors. While fever, leukocytosis and an elevated CRP are common in patients with TOA, about 40% of patients with TOA may have a normal temperature and leukocyte count, according to findings of the current study. Many studies have indicated that serum CA125 is a valuable tumor marker for predicting ovarian epithelial malignant tumors, however, increased serum CA125 has also been reported in endometriosis and inflammatory pelvic diseases in previous studies [18]. Our results indicated no significant differences in serum CA125 levels between the TOA and ovarian malignancy groups. We therefore consider CA125 elevation unreliable in differentiating pelvic malignant mass from TOA.

MRI usually has a high accuracy in diagnosis of tubo-ovarian or ovarian abscess with clinical pelvic inflammatory symptoms or signs and leukocytosis. An ill-defined adnexal mass with thick regular or irregular enhanced walls containing fluid is typical of MRI findings of TOA, with hypointensity on T1-weighted images and hyperintensity on T2-weighted images [19,20]. However, the morphology and signal intensities of TOA may alter greatly from its initiation, to its progression and degradation. The present study found that only a proportion of TOAs present with typical MRI findings, while the majority showed mixed cystic and solid mass. TOAs with obvious solid component mostly presented striking rim-enhancement on post-contrast images, pathologically proven to be the abundant granulation tissue and fibrous tissue hyperplasia peripherally [21]. Moreover, the signal intensities in abscess cavity varied from low signal intensity to intermediate or high signal intensity on T1/T2-weighted images with 82.4% of TOAs showing similar signal intensity to those of soft-tissue or mucinous components of adnexal masses. TOAs typically shows infiltrative inflammatory process, with indistinct margins and invasion into adjacent organs, mimicking the aggressive nature of ovarian malignancies. In the current study, we found that TOA frequently implicates the fallopian tube (salpingitis or pyosalpinx), while this is not common in ovarian cancer, which is consistent with the previous literature [1]. The multivariate logistic regression analysis also demonstrated that the imaging feature of dilated tubal structure was one of independent risk predictors for TOA. Differential diagnosis of TOA and ovarian malignancy can be challenging when clinical pelvic inflammatory symptoms or signs and leukocytosis are vague, and MRI imaging features are atypical.Therefore, when detecting a complex adnexal mass associated with dilated fallopian tubes, we may have more confidence in the diagnosis of TOA [3].

DWI is a functional imaging modality for assessing water diffusion, and its ADC value is a quantitative parameter for the diffusion coefficient. Recent years, DWI has been increasingly used in the evaluation of pelvic diseases, including both tumor and inflammatory diseases. Li et al. reported that the DW imaging with its ADC values can improve the characterization of clinically suspected PID [8]. Hyperintensity on DW imaging with low ADC value can be found in pus, highly viscous fluid, high-cellularity tumor and coagulative necrosis. Our study revealed that the fluid collections in the cavity of all 34 TOAs show high signal intensity on DWI with lower ADC values compared to the cystic component of ovarian malignancy. In the logistic regression analysis, it indicated that ADC value was another diagnostic predictors for TOA. The ROC curve analysis showed combination diagnosis of ADC value and dilated tubal structure achieved the best AUC of 0.996. Using ADC measurements, MRI combined with DWI can lead to correct diagnosis of TOA with an extremely high degree of accuracy, sensitivity and specificity, compared to MRI.

In previous studies, abscess cavities usually show very low ADC values, which reflect the initial ADC values [8,17]. Our results of the fluid collections in the TOA were a little higher than the initial ADC values of the abscess. The signal intensity on DWI and the corresponding ADC values of abscesses can change at different stages or alter after treatment with empiric antibiotics. At an early stage of pyogenesis, the pus doesn’t have a high viscosity and inflammatory cellularity. Holzapfel et al. reported that an early liver abscess had higher ADC values (1.2−1.6 × 10^−3^ mm^2^/s) than a mature abscess (0.3−0.7 × 10^−3^ mm^2^/s) [22]. As the inflammation subsides, the pus in a mature abscess gradually changes to thin serous fluid. For this reason, the abscess cavity sometimes shows low or intermediate signal intensity, with only a patchy of residual pus presenting high signal intensity on DW images.

Another previous study demonstrated that pelvic abscesses on DW-MRI could be easily detected without using intravenous contrast agents, while it is not applicable for those TOAs with pseudosolid areas[10]. In our study, 82.4% patients with TOA presented pseudosolid-like signal intensity on T1 and T2-weighted images with high signal intensity on DW images with low ADC values, which is similar to that of solid components of ovarian tumors. So we believe that it is difficult to separate solid parts of adnexal tumors from pus with pseudosolid-like signal intensity, only depending on signal characteristics on noncontrast MRI and DWI. After contrast injection, the pus of abscess reveal no enhancement, which can be easily distinguished from the enhenced solid parts of adnexal tumors.

Our findings demonstrated that TOA with enhanced solid components can mimic ovarian malignant tumor, especially those with high signal intensity on DWI (pseudotumor area). In our study, twelve patients with TOA showed high signal intensity in the solid component on DW-MRI, but with a higher ADC value than that of malignancy. There are overlaps between them. We speculate that with the progression of TOA, the formation of abundant granulation tissue and fibrous tissue hyperplasia more or less restrict the movement of water molecules, and inflammatory cytokines lead to local high microcirculation perfusion and the edema of cells, thus presenting high signal intensity on DWI. On the other hand, a few borderline tumors with relative free-moving water molecules were included in our study, which may in part explain why our results of solid ADC value in malignancy were higher than in previous studies [4,23].

The current study provides a thorough understanding of pseudotumor areas in TOA, which demonstrate soft-tissue or pseudosolid-like signal intensity on T1 and T2-weighted images, marked enhancement on post-contrast MR image, and hyperintensity on DWI with a relatively high ADC value. However, this preliminary study had some limitations. In the present retrospective study, a simplified mono exponential model of only two b values (b = 0 and 1000 s/mm^2^) was used, which is currently the most common protocol applied in clinical DWI systems with an adequate signal to noise ratio (SNR) [24,25]. However, the ADC calculated from only two b values may give an incomplete picture of the true diffusion process, thus limiting the accuracy of ADC calculations. Several recent studies have recommended using bi-exponential or more complex models with a set of b values, which can provide more exact information about both the diffusion and perfusion fraction in the tissues according to the intravoxel incoherent motion(IVIM) theory [26]. While more intermediate b values would be desirable, wide application of bi-exponential or more complex models in the pelvis have not to date been established, and further prospective studies are required to determine the optimal model for ADC determination. Secondly, the sample size was relatively small. All patients were highly selected, and only a few borderline tumors with relative free-moving water molecules were included in the study. This may in part explain why our results of solid ADC value in ovarian malignancy had overlaps with that of pseudotumor areas of TOA. However, some of the enrolled patients had antibiotic treatment before MRI scanning, and we did not classify the different stages of the TOA or confirm specific pathogenic organisms [11]. The presence and effect different pathogenic organisms, age of abscess, and treatment with empiric antibiotics might result in variations of signal intensity on DWI and ADC values, and should therefore be taken into consideration. As such, our results might not be generally applicable and require confirmation in further clinical studies that involve larger sample sizes.

## Conclusions

In conclusion, our results indicate that DW-MRI is superior to MRI in the accurate diagnosis of TOA mimicking ovarian malignancy, with extremely high accuracy, sensitivity, PPV and NPV. Furthermore, ADC values can provide more comprehensive information to distinguish the pseudotumor areas of TOAs from the solid portions of ovarian malignancy. Furthermore, the DW images and ADC values should be combined with MR imaging, to comprehensively analyze the corresponding cystic part or the enhanced solid part between TOA and malignancy. Future prospective studies involving larger sample sizes, are necessary to systematically validate our findings.
